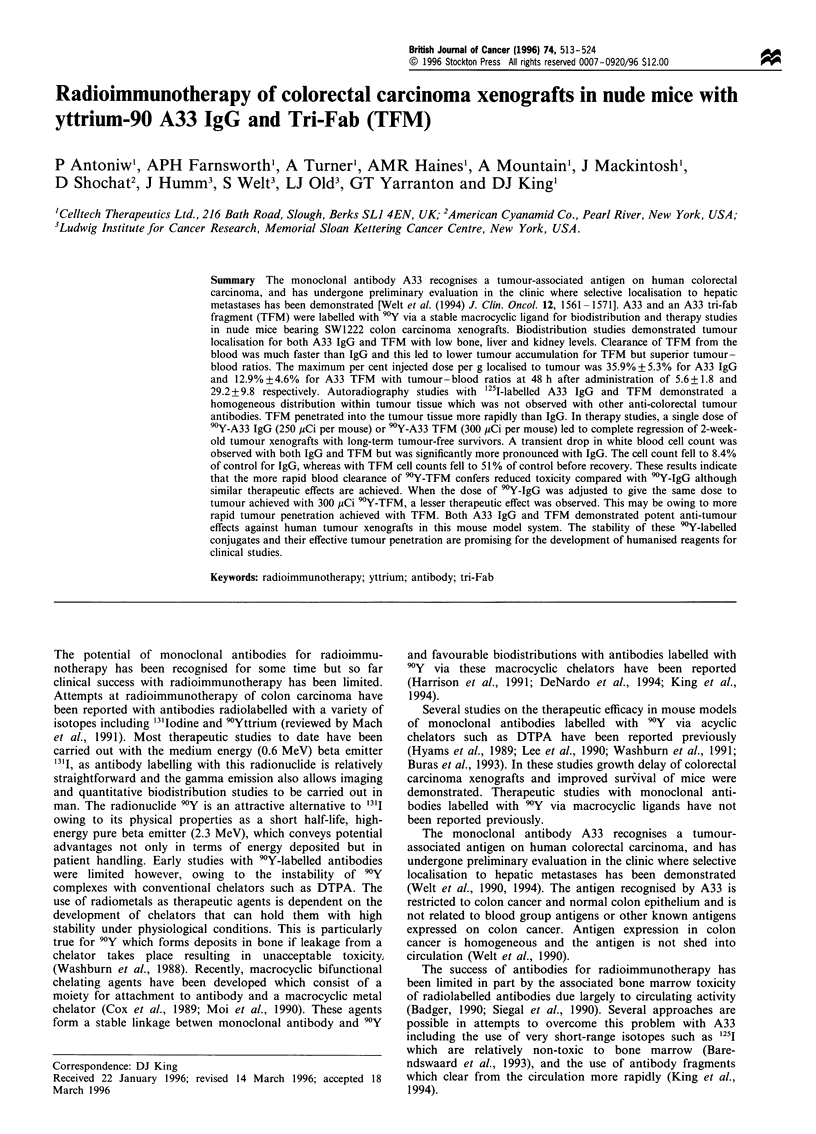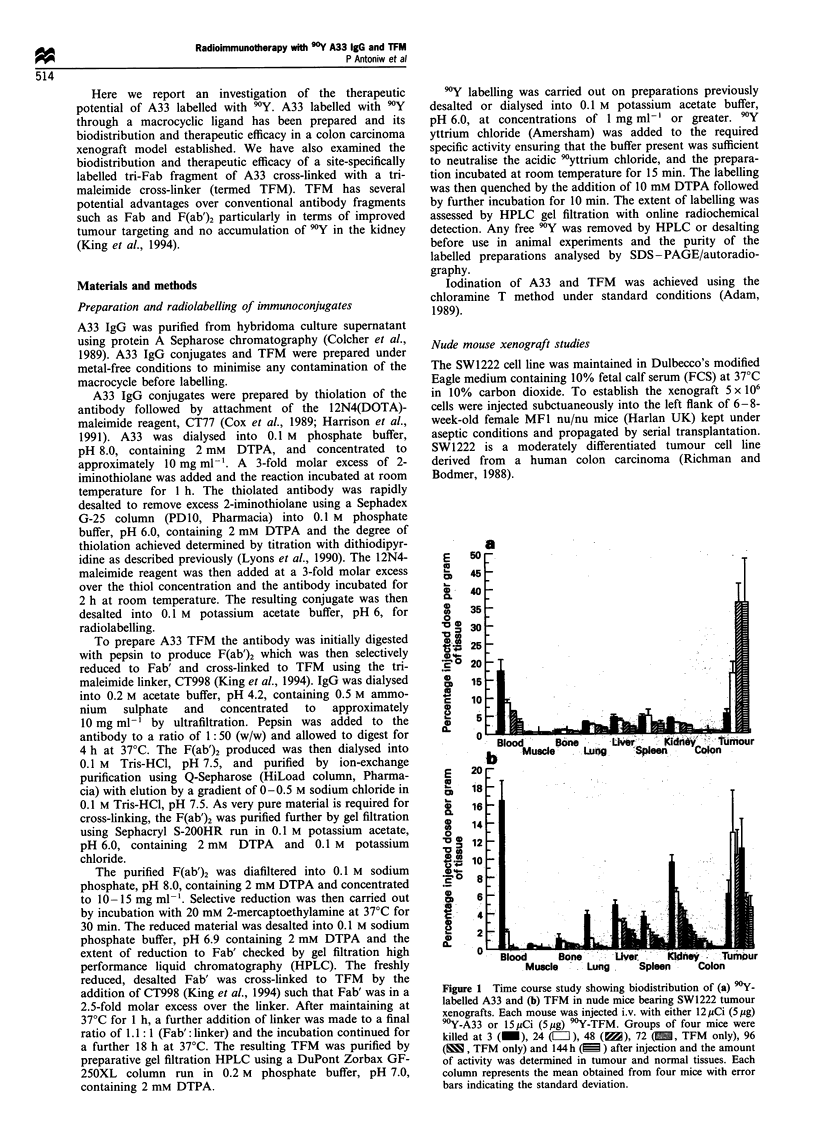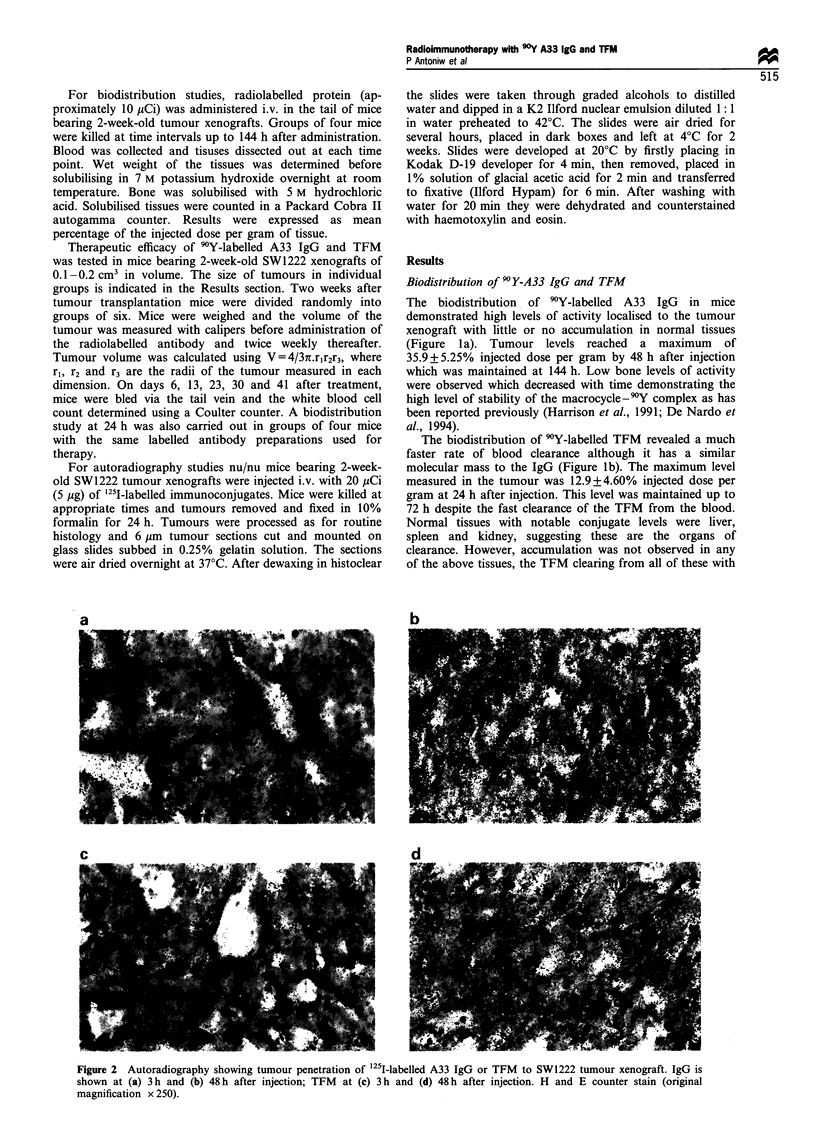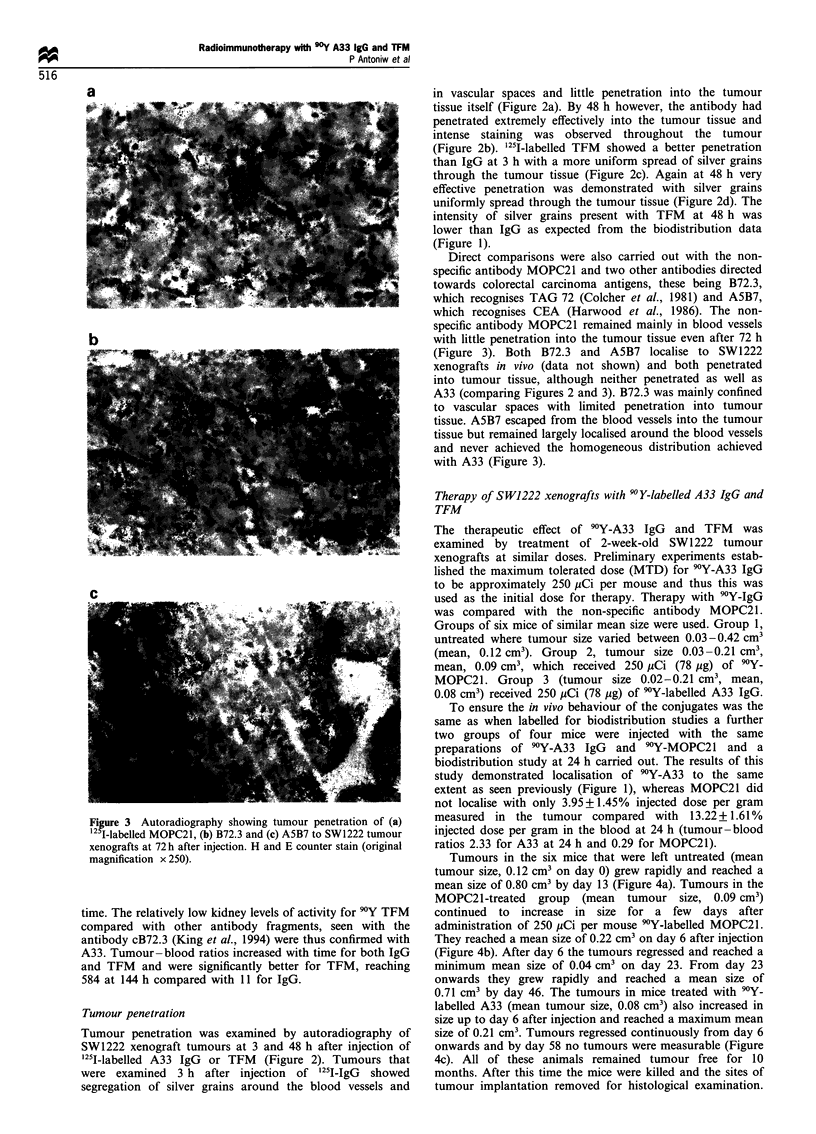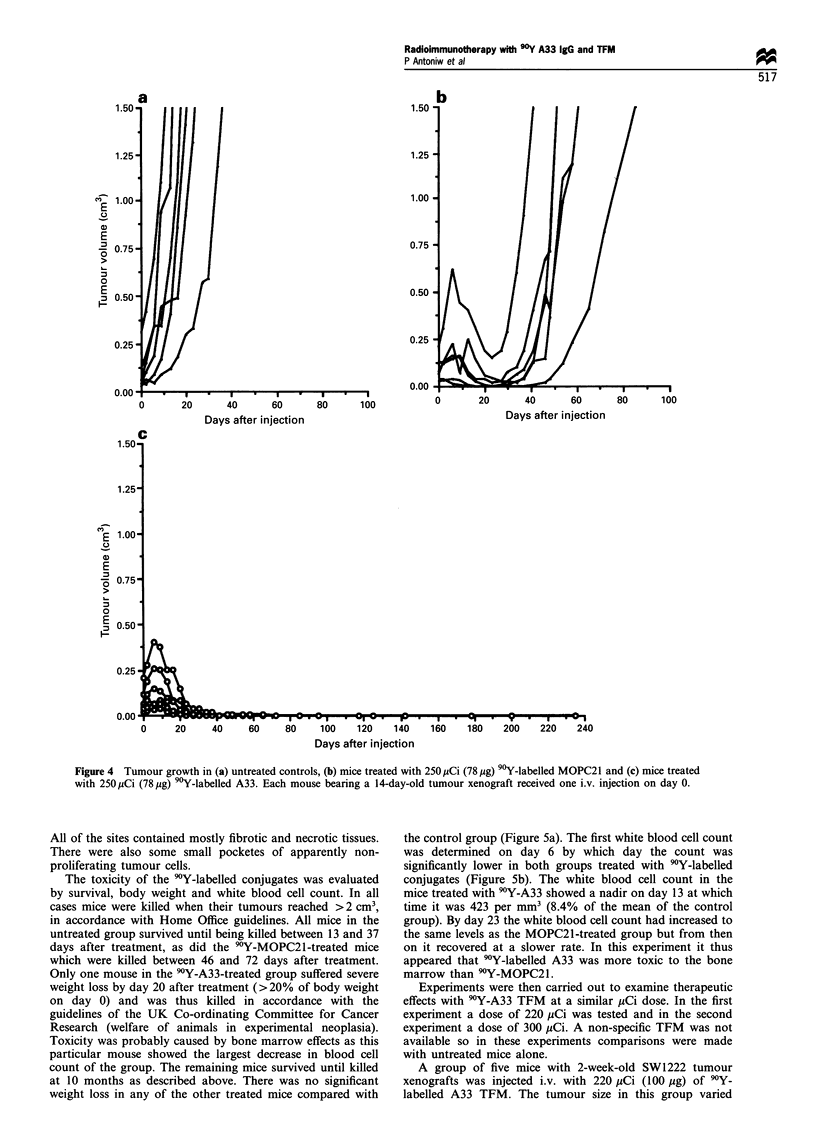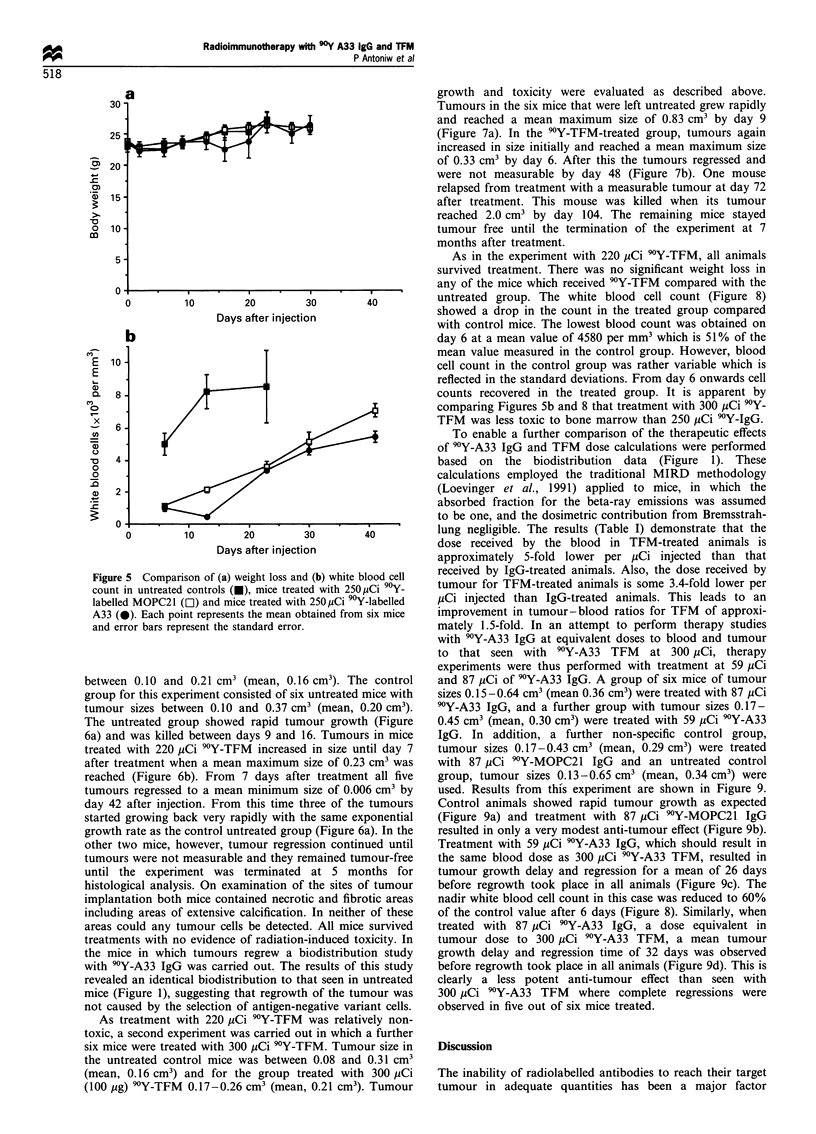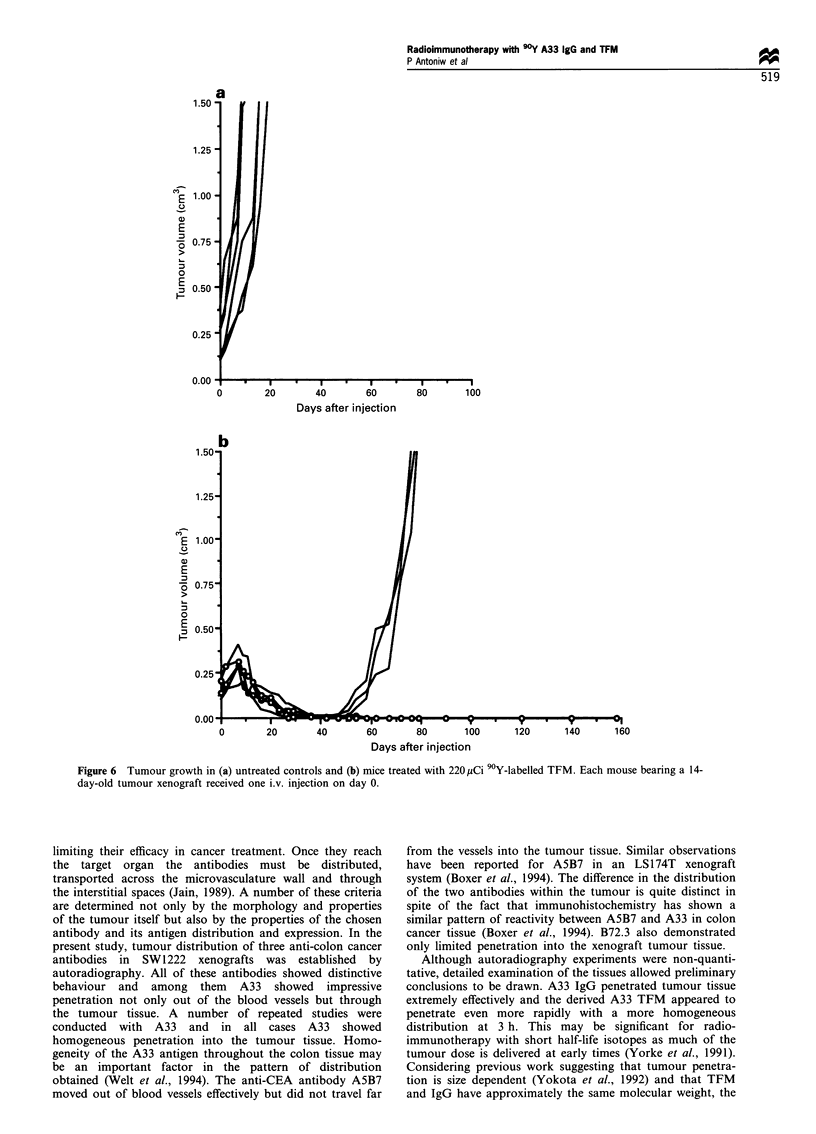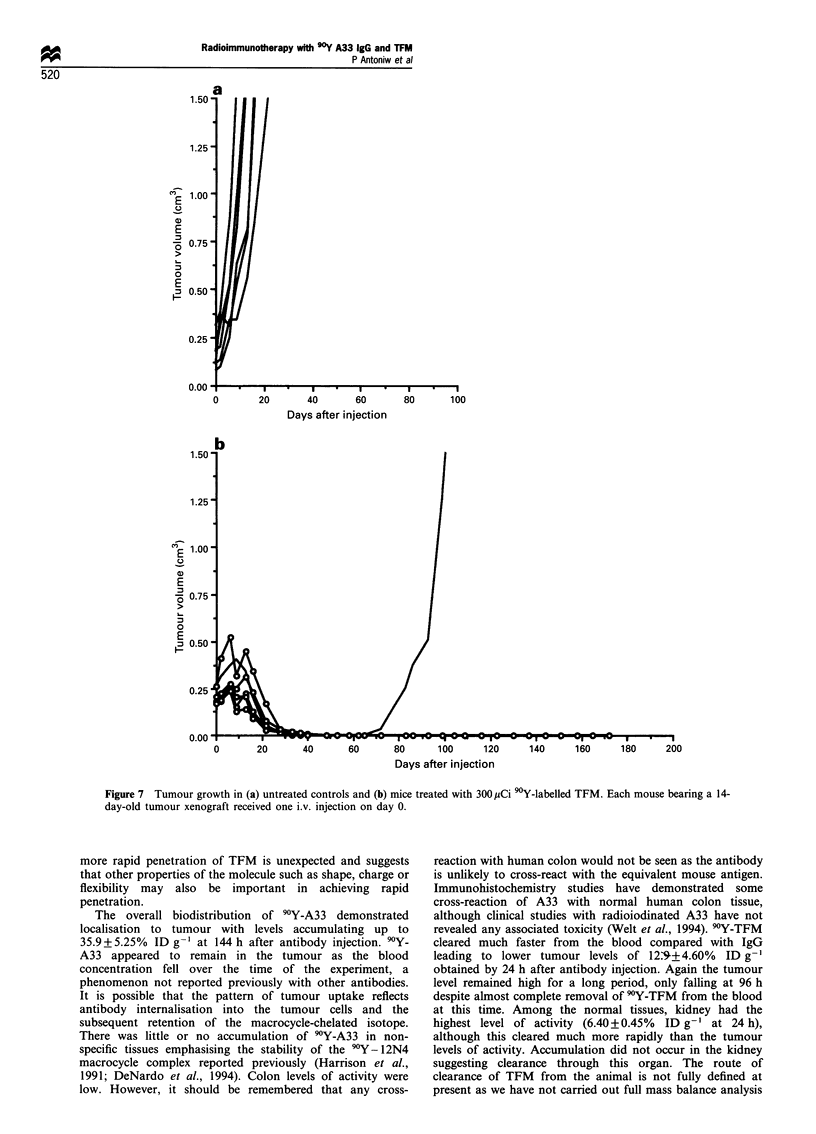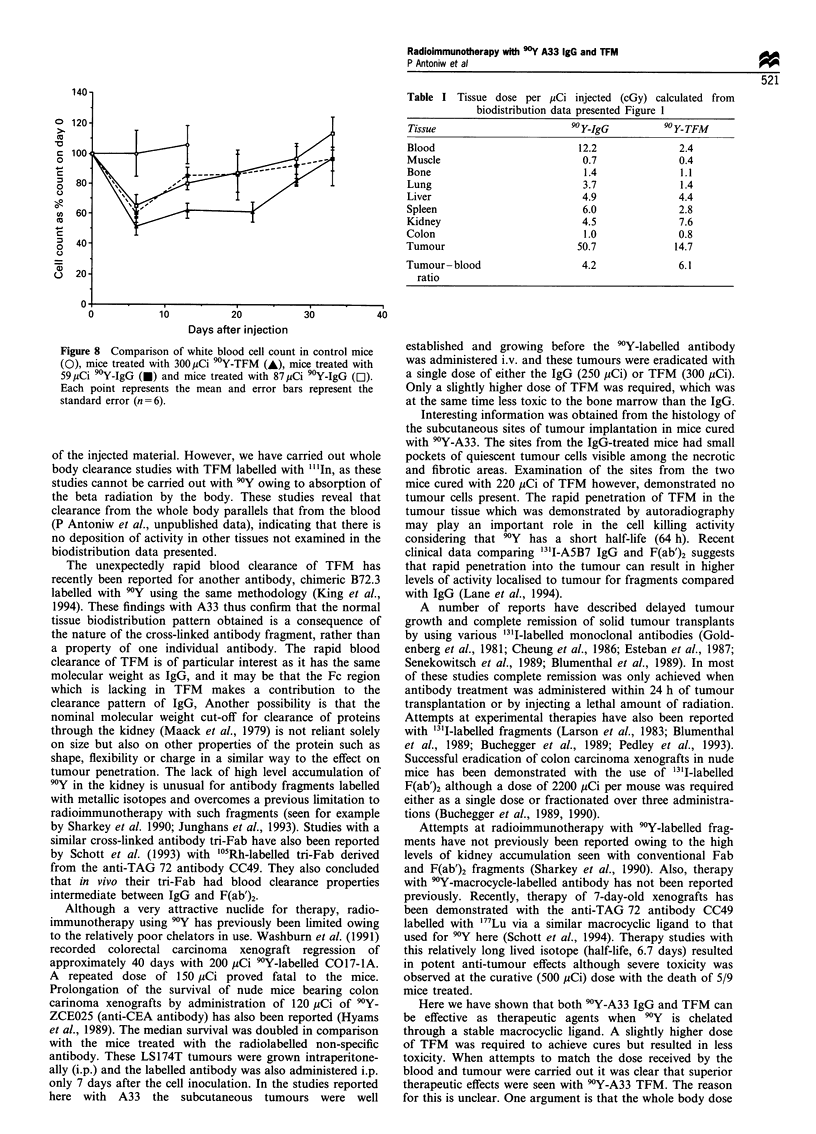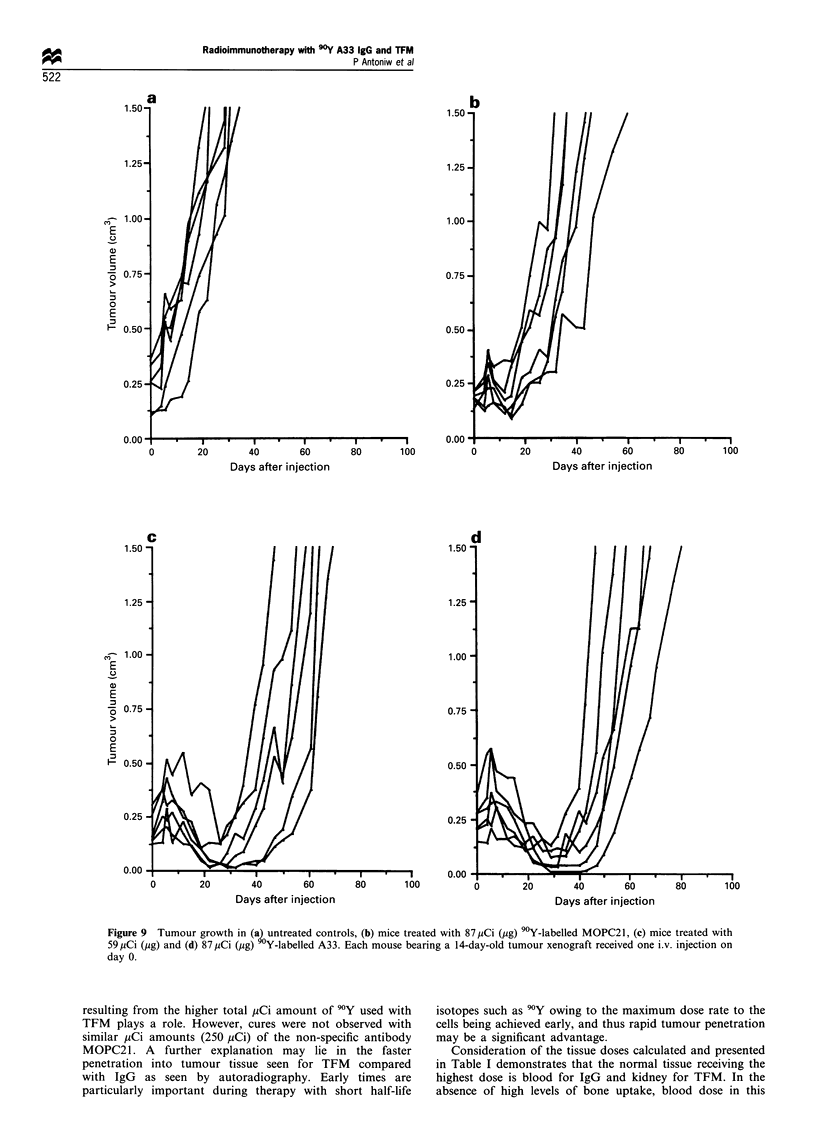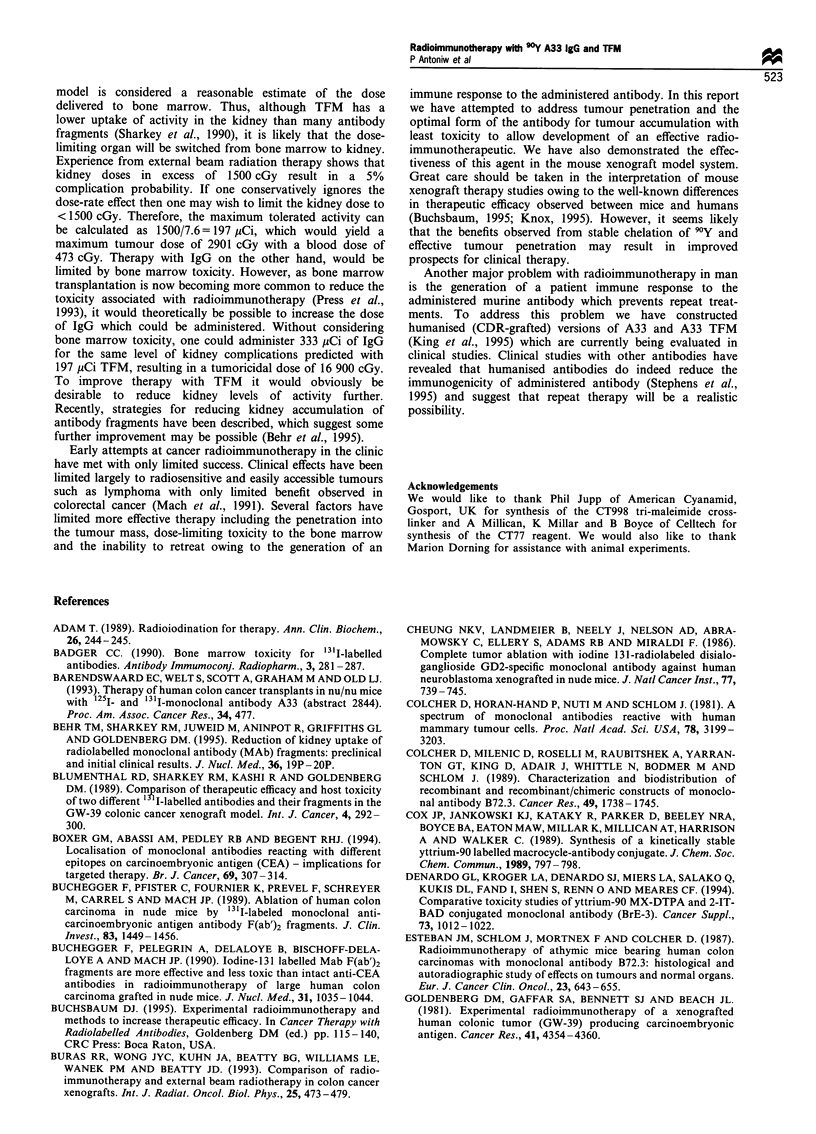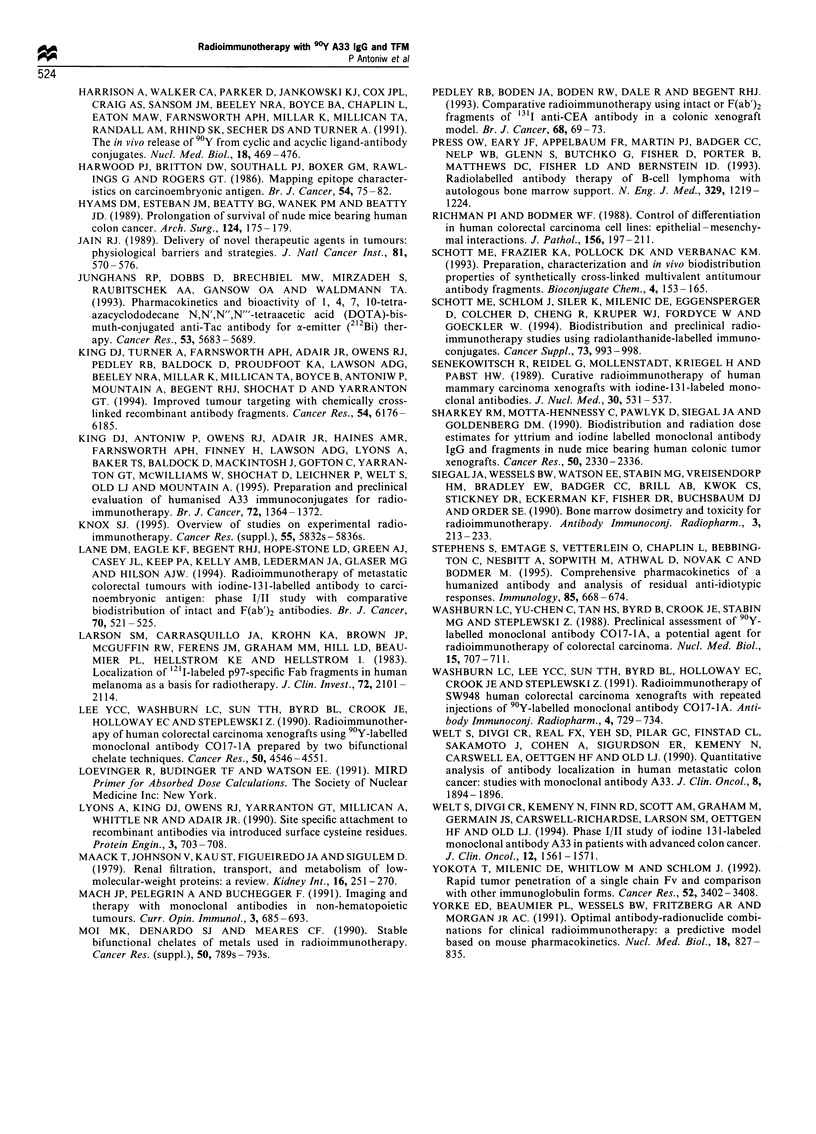# Radioimmunotherapy of colorectal carcinoma xenografts in nude mice with yttrium-90 A33 IgG and Tri-Fab (TFM).

**DOI:** 10.1038/bjc.1996.395

**Published:** 1996-08

**Authors:** P. Antoniw, A. P. Farnsworth, A. Turner, A. M. Haines, A. Mountain, J. Mackintosh, D. Shochat, J. Humm, S. Welt, L. J. Old, G. T. Yarranton, D. J. King

**Affiliations:** Celltech Therapeutics Ltd., Slough, Berks, UK.

## Abstract

**Images:**


British Journal of Cancer (1996) 74, 513-524
© 1996 Stockton Press All rights reserved 0007-0920/96 $12.00

### Radioimmunotherapy of colorectal carcinoma xenografts in nude mice with yttrium-90 A33 IgG and Tri-Fab (TFM)

P Antoniw[1], APH Farnsworth[1], A Turner[1], AMR Haines[1], A Mountain[1], J Mackintosh[1], D Shochat[2], J Humm[3], S Welt[3], LJ Old[3], GT Yarranton and DJ King[1]

[1]*Celltech Therapeutics Ltd., 216 Bath Road, Slough, Berks SL1 4EN, UK;* [2]*American Cyanamid Co., Pearl River, New York, USA;* [3]*Ludwig Institute for Cancer Research, Memorial Sloan Kettering Cancer Centre, New York, USA.*

**Summary** The monoclonal antibody A33 recognises a tumour-associated antigen on human colorectal carcinoma, and has undergone preliminary evaluation in the clinic where selective localisation to hepatic metastases has been demonstrated [Welt *et al.* (1994) *J. Clin. Oncol.* **12**, 1561-1571]. A33 and an A33 tri-fab fragment (TFM) were labelled with $^{90}$Y via a stable macrocyclic ligand for biodistribution and therapy studies in nude mice bearing SW1222 colon carcinoma xenografts. Biodistribution studies demonstrated tumour localisation for both A33 IgG and TFM with low bone, liver and kidney levels. Clearance of TFM from the blood was much faster than IgG and this led to lower tumour accumulation for TFM but superior tumour-blood ratios. The maximum per cent injected dose per g localised to tumour was 35.9% $\pm$5.3% for A33 IgG and 12.9% $\pm$4.6% for A33 TFM with tumour-blood ratios at 48 h after administration of 5.6 $\pm$ 1.8 and 29.2 $\pm$ 9.8 respectively. Autoradiography studies with $^{125}$I-labelled A33 IgG and TFM demonstrated a homogeneous distribution within tumour tissue which was not observed with other anti-colorectal tumour antibodies. TFM penetrated into the tumour tissue more rapidly than IgG. In therapy studies, a single dose of $^{90}$Y-A33 IgG (250 $\mu$Ci per mouse) or $^{90}$Y-A33 TFM (300 $\mu$Ci per mouse) led to complete regression of 2-week-old tumour xenografts with long-term tumour-free survivors. A transient drop in white blood cell count was observed with both IgG and TFM but was significantly more pronounced with IgG. The cell count fell to 8.4% of control for IgG, whereas with TFM cell counts fell to 51% of control before recovery. These results indicate that the more rapid blood clearance of $^{90}$Y-TFM confers reduced toxicity compared with $^{90}$Y-IgG although similar therapeutic effects are achieved. When the dose of $^{90}$Y-IgG was adjusted to give the same dose to tumour achieved with 300 $\mu$Ci $^{90}$Y-TFM, a lesser therapeutic effect was observed. This may be owing to more rapid tumour penetration achieved with TFM. Both A33 IgG and TFM demonstrated potent anti-tumour effects against human tumour xenografts in this mouse model system. The stability of these $^{90}$Y-labelled conjugates and their effective tumour penetration are promising for the development of humanised reagents for clinical studies.

**Keywords:** radioimmunotherapy; yttrium; antibody; tri-Fab

The potential of monoclonal antibodies for radioimmunotherapy has been recognised for some time but so far clinical success with radioimmunotherapy has been limited. Attempts at radioimmunotherapy of colon carcinoma have been reported with antibodies radiolabelled with a variety of isotopes including $^{131}$Iodine and $^{90}$Yttrium (reviewed by Mach *et al.*, 1991). Most therapeutic studies to date have been carried out with the medium energy (0.6 MeV) beta emitter $^{131}$I, as antibody labelling with this radionuclide is relatively straightforward and the gamma emission also allows imaging and quantitative biodistribution studies to be carried out in man. The radionuclide $^{90}$Y is an attractive alternative to $^{131}$I owing to its physical properties as a short half-life, high-energy pure beta emitter (2.3 MeV), which conveys potential advantages not only in terms of energy deposited but in patient handling. Early studies with $^{90}$Y-labelled antibodies were limited however, owing to the instability of $^{90}$Y complexes with conventional chelators such as DTPA. The use of radiometals as therapeutic agents is dependent on the development of chelators that can hold them with high stability under physiological conditions. This is particularly true for $^{90}$Y which forms deposits in bone if leakage from a chelator takes place resulting in unacceptable toxicity (Washburn *et al.*, 1988). Recently, macrocyclic bifunctional chelating agents have been developed which consist of a moiety for attachment to antibody and a macrocyclic metal chelator (Cox *et al.*, 1989; Moi *et al.*, 1990). These agents form a stable linkage betwen monoclonal antibody and $^{90}$Y and favourable biodistributions with antibodies labelled with $^{90}$Y via these macrocyclic chelators have been reported (Harrison *et al.*, 1991; DeNardo *et al.*, 1994; King *et al.*, 1994).

Several studies on the therapeutic efficacy in mouse models of monoclonal antibodies labelled with $^{90}$Y via acyclic chelators such as DTPA have been reported previously (Hyams *et al.*, 1989; Lee *et al.*, 1990; Washburn *et al.*, 1991; Buras *et al.*, 1993). In these studies growth delay of colorectal carcinoma xenografts and improved survival of mice were demonstrated. Therapeutic studies with monoclonal antibodies labelled with $^{90}$Y via macrocyclic ligands have not been reported previously.

The monoclonal antibody A33 recognises a tumour-associated antigen on human colorectal carcinoma, and has undergone preliminary evaluation in the clinic where selective localisation to hepatic metastases has been demonstrated (Welt *et al.*, 1990, 1994). The antigen recognised by A33 is restricted to colon cancer and normal colon epithelium and is not related to blood group antigens or other known antigens expressed on colon cancer. Antigen expression in colon cancer is homogeneous and the antigen is not shed into circulation (Welt *et al.*, 1990).

The success of antibodies for radioimmunotherapy has been limited in part by the associated bone marrow toxicity of radiolabelled antibodies due largely to circulating activity (Badger, 1990; Siegal *et al.*, 1990). Several approaches are possible in attempts to overcome this problem with A33 including the use of very short-range isotopes such as $^{125}$I which are relatively non-toxic to bone marrow (Barendswaard *et al.*, 1993), and the use of antibody fragments which clear from the circulation more rapidly (King *et al.*, 1994).

Correspondence: DJ King
Received 22 January 1996; revised 14 March 1996; accepted 18 March 1996

Here we report an investigation of the therapeutic potential of A33 labelled with $^{90}$Y. A33 labelled with $^{90}$Y through a macrocyclic ligand has been prepared and its biodistribution and therapeutic efficacy in a colon carcinoma xenograft model established. We have also examined the biodistribution and therapeutic efficacy of a site-specifically labelled tri-Fab fragment of A33 cross-linked with a tri-maleimide cross-linker (termed TFM). TFM has several potential advantages over conventional antibody fragments such as Fab and F(ab')$_2$ particularly in terms of improved tumour targeting and no accumulation of $^{90}$Y in the kidney (King *et al.*, 1994).

### Materials and methods

### *Preparation and radiolabelling of immunoconjugates*

A33 IgG was purified from hybridoma culture supernatant using protein A Sepharose chromatography (Colcher *et al.*, 1989). A33 IgG conjugates and TFM were prepared under metal-free conditions to minimise any contamination of the macrocycle before labelling.

A33 IgG conjugates were prepared by thiolation of the antibody followed by attachment of the 12N4(DOTA)-maleimide reagent, CT77 (Cox *et al.*, 1989; Harrison *et al.*, 1991). A33 was dialysed into 0.1 M phosphate buffer, pH 8.0, containing 2 mM DTPA, and concentrated to approximately 10 mg ml$^{-1}$. A 3-fold molar excess of 2-iminothiolane was added and the reaction incubated at room temperature for 1 h. The thiolated antibody was rapidly desalted to remove excess 2-iminothiolane using a Sephadex G-25 column (PD10, Pharmacia) into 0.1 M phosphate buffer, pH 6.0, containing 2 mM DTPA and the degree of thiolation achieved determined by titration with dithiodipyridine as described previously (Lyons *et al.*, 1990). The 12N4-maleimide reagent was then added at a 3-fold molar excess over the thiol concentration and the antibody incubated for 2 h at room temperature. The resulting conjugate was then desalted into 0.1 M potassium acetate buffer, pH 6, for radiolabelling.

To prepare A33 TFM the antibody was initially digested with pepsin to produce F(ab')$_2$ which was then selectively reduced to Fab' and cross-linked to TFM using the tri-maleimide linker, CT998 (King *et al.*, 1994). IgG was dialysed into 0.2 M acetate buffer, pH 4.2, containing 0.5 M ammonium sulphate and concentrated to approximately 10 mg ml$^{-1}$ by ultrafiltration. Pepsin was added to the antibody to a ratio of 1 : 50 (w/w) and allowed to digest for 4 h at 37°C. The F(ab')$_2$ produced was then dialysed into 0.1 M Tris-HCl, pH 7.5, and purified by ion-exchange purification using Q-Sepharose (HiLoad column, Pharmacia) with elution by a gradient of 0–0.5 M sodium chloride in 0.1 M Tris-HCl, pH 7.5. As very pure material is required for cross-linking, the F(ab')$_2$ was purified further by gel filtration using Sephacryl S-200HR run in 0.1 M potassium acetate, pH 6.0, containing 2 mM DTPA and 0.1 M potassium chloride.

The purified F(ab')$_2$ was diafiltered into 0.1 M sodium phosphate, pH 8.0, containing 2 mM DTPA and concentrated to 10–15 mg ml$^{-1}$. Selective reduction was then carried out by incubation with 20 mM 2-mercaptoethylamine at 37°C for 30 min. The reduced material was desalted into 0.1 M sodium phosphate buffer, pH 6.9 containing 2 mM DTPA and the extent of reduction to Fab' checked by gel filtration high performance liquid chromatography (HPLC). The freshly reduced, desalted Fab' was cross-linked to TFM by the addition of CT998 (King *et al.*, 1994) such that Fab' was in a 2.5-fold molar excess over the linker. After maintaining at 37°C for 1 h, a further addition of linker was made to a final ratio of 1.1 : 1 (Fab' : linker) and the incubation continued for a further 18 h at 37°C. The resulting TFM was purified by preparative gel filtration HPLC using a DuPont Zorbax GF-250XL column run in 0.2 M phosphate buffer, pH 7.0, containing 2 mM DTPA.

$^{90}$Y labelling was carried out on preparations previously desalted or dialysed into 0.1 M potassium acetate buffer, pH 6.0, at concentrations of 1 mg ml$^{-1}$ or greater. $^{90}$Y yttrium chloride (Amersham) was added to the required specific activity ensuring that the buffer present was sufficient to neutralise the acidic $^{90}$yttrium chloride, and the preparation incubated at room temperature for 15 min. The labelling was then quenched by the addition of 10 mM DTPA followed by further incubation for 10 min. The extent of labelling was assessed by HPLC gel filtration with online radiochemical detection. Any free $^{90}$Y was removed by HPLC or desalting before use in animal experiments and the purity of the labelled preparations analysed by SDS–PAGE/autoradiography.

Iodination of A33 and TFM was achieved using the chloramine T method under standard conditions (Adam, 1989).

### *Nude mouse xenograft studies*

The SW1222 cell line was maintained in Dulbecco's modified Eagle medium containing 10% fetal calf serum (FCS) at 37°C in 10% carbon dioxide. To establish the xenograft $5 \times 10^6$ cells were injected subctuaneously into the left flank of 6–8-week-old female MF1 nu/nu mice (Harlan UK) kept under aseptic conditions and propagated by serial transplantation. SW1222 is a moderately differentiated tumour cell line derived from a human colon carcinoma (Richman and Bodmer, 1988).

**Figure 1** Time course study showing biodistribution of (**a**) $^{90}$Y-labelled A33 and (**b**) TFM in nude mice bearing SW1222 tumour xenografts. Each mouse was injected i.v. with either 12 $\mu$Ci (5 $\mu$g) $^{90}$Y-A33 or 15 $\mu$Ci (5 $\mu$g) $^{90}$Y-TFM. Groups of four mice were killed at 3 (■), 24 (□), 48 (▨), 72 (▩, TFM only), 96 (▧, TFM only) and 144 h (▤) after injection and the amount of activity was determined in tumour and normal tissues. Each column represents the mean obtained from four mice with error bars indicating the standard deviation.

For biodistribution studies, radiolabelled protein (approximately 10 $\mu$Ci) was administered i.v. in the tail of mice bearing 2-week-old tumour xenografts. Groups of four mice were killed at time intervals up to 144 h after administration. Blood was collected and tisuses dissected out at each time point. Wet weight of the tissues was determined before solubilising in 7 M potassium hydroxide overnight at room temperature. Bone was solubilised with 5 M hydrochloric acid. Solubilised tissues were counted in a Packard Cobra II autogamma counter. Results were expressed as mean percentage of the injected dose per gram of tissue.

Therapeutic efficacy of $^{90}$Y-labelled A33 IgG and TFM was tested in mice bearing 2-week-old SW1222 xenografts of 0.1 – 0.2 $cm^3$ in volume. The size of tumours in individual groups is indicated in the Results section. Two weeks after tumour transplantation mice were divided randomly into groups of six. Mice were weighed and the volume of the tumour was measured with calipers before administration of the radiolabelled antibody and twice weekly thereafter. Tumour volume was calculated using $V = 4/3\pi.r_1r_2r_3$, where $r_1$, $r_2$ and $r_3$ are the radii of the tumour measured in each dimension. On days 6, 13, 23, 30 and 41 after treatment, mice were bled via the tail vein and the white blood cell count determined using a Coulter counter. A biodistribution study at 24 h was also carried out in groups of four mice with the same labelled antibody preparations used for therapy.

For autoradiography studies nu/nu mice bearing 2-week-old SW1222 tumour xenografts were injected i.v. with 20 $\mu$Ci (5 $\mu$g) of $^{125}$I-labelled immunoconjugates. Mice were killed at appropriate times and tumours removed and fixed in 10% formalin for 24 h. Tumours were processed as for routine histology and 6 $\mu$m tumour sections cut and mounted on glass slides subbed in 0.25% gelatin solution. The sections were air dried overnight at 37°C. After dewaxing in histoclear the slides were taken through graded alcohols to distilled water and dipped in a K2 Ilford nuclear emulsion diluted 1 : 1 in water preheated to 42°C. The slides were air dried for several hours, placed in dark boxes and left at 4°C for 2 weeks. Slides were developed at 20°C by firstly placing in Kodak D-19 developer for 4 min, then removed, placed in 1% solution of glacial acetic acid for 2 min and transferred to fixative (Ilford Hypam) for 6 min. After washing with water for 20 min they were dehydrated and counterstained with haemotoxylin and eosin.

### Results

### *Biodistribution of $^{90}$Y-A33 IgG and TFM*

The biodistribution of $^{90}$Y-labelled A33 IgG in mice demonstrated high levels of activity localised to the tumour xenograft with little or no accumulation in normal tissues (Figure 1a). Tumour levels reached a maximum of 35.9 ± 5.25% injected dose per gram by 48 h after injection which was maintained at 144 h. Low bone levels of activity were observed which decreased with time demonstrating the high level of stability of the macrocycle – $^{90}$Y complex as has been reported previously (Harrison *et al*., 1991; De Nardo *et al*., 1994).

The biodistribution of $^{90}$Y-labelled TFM revealed a much faster rate of blood clearance although it has a similar molecular mass to the IgG (Figure 1b). The maximum level measured in the tumour was 12.9 ± 4.60% injected dose per gram at 24 h after injection. This level was maintained up to 72 h despite the fast clearance of the TFM from the blood. Normal tissues with notable conjugate levels were liver, spleen and kidney, suggesting these are the organs of clearance. However, accumulation was not observed in any of the above tissues, the TFM clearing from all of these with

**Figure 2** Autoradiography showing tumour penetration of $^{125}$I-labelled A33 IgG or TFM to SW1222 tumour xenograft. IgG is shown at (**a**) 3 h and (**b**) 48 h after injection; TFM at (**c**) 3 h and (**d**) 48 h after injection. H and E counter stain (original magnification ×250).

**Figure 3** Autoradiography showing tumour penetration of (**a**) $^{125}$I-labelled MOPC21, (**b**) B72.3 and (**c**) A5B7 to SW1222 tumour xenografts at 72 h after injection. H and E counter stain (original magnification $\times$250).

time. The relatively low kidney levels of activity for $^{90}$Y TFM compared with other antibody fragments, seen with the antibody cB72.3 (King *et al*., 1994) were thus confirmed with A33. Tumour–blood ratios increased with time for both IgG and TFM and were significantly better for TFM, reaching 584 at 144 h compared with 11 for IgG.

*Tumour penetration*

Tumour penetration was examined by autoradiography of SW1222 xenograft tumours at 3 and 48 h after injection of $^{125}$I-labelled A33 IgG or TFM (Figure 2). Tumours that were examined 3 h after injection of $^{125}$I-IgG showed segregation of silver grains around the blood vessels and in vascular spaces and little penetration into the tumour tissue itself (Figure 2a). By 48 h however, the antibody had penetrated extremely effectively into the tumour tissue and intense staining was observed throughout the tumour (Figure 2b). $^{125}$I-labelled TFM showed a better penetration than IgG at 3 h with a more uniform spread of silver grains through the tumour tissue (Figure 2c). Again at 48 h very effective penetration was demonstrated with silver grains uniformly spread through the tumour tissue (Figure 2d). The intensity of silver grains present with TFM at 48 h was lower than IgG as expected from the biodistribution data (Figure 1).

Direct comparisons were also carried out with the non-specific antibody MOPC21 and two other antibodies directed towards colorectal carcinoma antigens, these being B72.3, which recognises TAG 72 (Colcher *et al*., 1981) and A5B7, which recognises CEA (Harwood *et al*., 1986). The non-specific antibody MOPC21 remained mainly in blood vessels with little penetration into the tumour tissue even after 72 h (Figure 3). Both B72.3 and A5B7 localise to SW1222 xenografts *in vivo* (data not shown) and both penetrated into tumour tissue, although neither penetrated as well as A33 (comparing Figures 2 and 3). B72.3 was mainly confined to vascular spaces with limited penetration into tumour tissue. A5B7 escaped from the blood vessels into the tumour tissue but remained largely localised around the blood vessels and never achieved the homogeneous distribution achieved with A33 (Figure 3).

*Therapy of SW1222 xenografts with $^{90}$Y-labelled A33 IgG and TFM*

The therapeutic effect of $^{90}$Y-A33 IgG and TFM was examined by treatment of 2-week-old SW1222 tumour xenografts at similar doses. Preliminary experiments established the maximum tolerated dose (MTD) for $^{90}$Y-A33 IgG to be approximately 250 $\mu$Ci per mouse and thus this was used as the initial dose for therapy. Therapy with $^{90}$Y-IgG was compared with the non-specific antibody MOPC21. Groups of six mice of similar mean size were used. Group 1, untreated where tumour size varied between 0.03–0.42 cm$^3$ (mean, 0.12 cm$^3$). Group 2, tumour size 0.03–0.21 cm$^3$, mean, 0.09 cm$^3$, which received 250 $\mu$Ci (78 $\mu$g) of $^{90}$Y-MOPC21. Group 3 (tumour size 0.02–0.21 cm$^3$, mean, 0.08 cm$^3$) received 250 $\mu$Ci (78 $\mu$g) of $^{90}$Y-labelled A33 IgG.

To ensure the *in vivo* behaviour of the conjugates was the same as when labelled for biodistribution studies a further two groups of four mice were injected with the same preparations of $^{90}$Y-A33 IgG and $^{90}$Y-MOPC21 and a biodistribution study at 24 h carried out. The results of this study demonstrated localisation of $^{90}$Y-A33 to the same extent as seen previously (Figure 1), whereas MOPC21 did not localise with only 3.95$\pm$1.45% injected dose per gram measured in the tumour compared with 13.22$\pm$1.61% injected dose per gram in the blood at 24 h (tumour–blood ratios 2.33 for A33 at 24 h and 0.29 for MOPC21).

Tumours in the six mice that were left untreated (mean tumour size, 0.12 cm$^3$ on day 0) grew rapidly and reached a mean size of 0.80 cm$^3$ by day 13 (Figure 4a). Tumours in the MOPC21-treated group (mean tumour size, 0.09 cm$^3$) continued to increase in size for a few days after administration of 250 $\mu$Ci per mouse $^{90}$Y-labelled MOPC21. They reached a mean size of 0.22 cm$^3$ on day 6 after injection (Figure 4b). After day 6 the tumours regressed and reached a minimum mean size of 0.04 cm$^3$ on day 23. From day 23 onwards they grew rapidly and reached a mean size of 0.71 cm$^3$ by day 46. The tumours in mice treated with $^{90}$Y-labelled A33 (mean tumour size, 0.08 cm$^3$) also increased in size up to day 6 after injection and reached a maximum mean size of 0.21 cm$^3$. Tumours regressed continuously from day 6 onwards and by day 58 no tumours were measurable (Figure 4c). All of these animals remained tumour free for 10 months. After this time the mice were killed and the sites of tumour implantation removed for histological examination.

**Figure 4** Tumour growth in (a) untreated controls, (b) mice treated with 250 μCi (78 μg) $^{90}$Y-labelled MOPC21 and (c) mice treated with 250 μCi (78 μg) $^{90}$Y-labelled A33. Each mouse bearing a 14-day-old tumour xenograft received one i.v. injection on day 0.

All of the sites contained mostly fibrotic and necrotic tissues. There were also some small pocketes of apparently non-proliferating tumour cells.

The toxicity of the $^{90}$Y-labelled conjugates was evaluated by survival, body weight and white blood cell count. In all cases mice were killed when their tumours reached >2 cm$^3$, in accordance with Home Office guidelines. All mice in the untreated group survived until being killed between 13 and 37 days after treatment, as did the $^{90}$Y-MOPC21-treated mice which were killed between 46 and 72 days after treatment. Only one mouse in the $^{90}$Y-A33-treated group suffered severe weight loss by day 20 after treatment (>20% of body weight on day 0) and was thus killed in accordance with the guidelines of the UK Co-ordinating Committee for Cancer Research (welfare of animals in experimental neoplasia). Toxicity was probably caused by bone marrow effects as this particular mouse showed the largest decrease in blood cell count of the group. The remaining mice survived until killed at 10 months as described above. There was no significant weight loss in any of the other treated mice compared with the control group (Figure 5a). The first white blood cell count was determined on day 6 by which day the count was significantly lower in both groups treated with $^{90}$Y-labelled conjugates (Figure 5b). The white blood cell count in the mice treated with $^{90}$Y-A33 showed a nadir on day 13 at which time it was 423 per mm$^3$ (8.4% of the mean of the control group). By day 23 the white blood cell count had increased to the same levels as the MOPC21-treated group but from then on it recovered at a slower rate. In this experiment it thus appeared that $^{90}$Y-labelled A33 was more toxic to the bone marrow than $^{90}$Y-MOPC21.

Experiments were then carried out to examine therapeutic effects with $^{90}$Y-A33 TFM at a similar μCi dose. In the first experiment a dose of 220 μCi was tested and in the second experiment a dose of 300 μCi. A non-specific TFM was not available so in these experiments comparisons were made with untreated mice alone.

A group of five mice with 2-week-old SW1222 tumour xenografts was injected i.v. with 220 μCi (100 μg) of $^{90}$Y-labelled A33 TFM. The tumour size in this group varied

**Figure 5** Comparison of (**a**) weight loss and (**b**) white blood cell count in untreated controls (■), mice treated with 250 μCi $^{90}$Y-labelled MOPC21 (□) and mice treated with 250 μCi $^{90}$Y-labelled A33 (●). Each point represents the mean obtained from six mice and error bars represent the standard error.

between 0.10 and 0.21 cm$^3$ (mean, 0.16 cm$^3$). The control group for this experiment consisted of six untreated mice with tumour sizes between 0.10 and 0.37 cm$^3$ (mean, 0.20 cm$^3$). The untreated group showed rapid tumour growth (Figure 6a) and was killed between days 9 and 16. Tumours in mice treated with 220 μCi $^{90}$Y-TFM increased in size until day 7 after treatment when a mean maximum size of 0.23 cm$^3$ was reached (Figure 6b). From 7 days after treatment all five tumours regressed to a mean minimum size of 0.006 cm$^3$ by day 42 after injection. From this time three of the tumours started growing back very rapidly with the same exponential growth rate as the control untreated group (Figure 6a). In the other two mice, however, tumour regression continued until tumours were not measurable and they remained tumour-free until the experiment was terminated at 5 months for histological analysis. On examination of the sites of tumour implantation both mice contained necrotic and fibrotic areas including areas of extensive calcification. In neither of these areas could any tumour cells be detected. All mice survived treatments with no evidence of radiation-induced toxicity. In the mice in which tumours regrew a biodistribution study with $^{90}$Y-A33 IgG was carried out. The results of this study revealed an identical biodistribution to that seen in untreated mice (Figure 1), suggesting that regrowth of the tumour was not caused by the selection of antigen-negative variant cells.

As treatment with 220 μCi $^{90}$Y-TFM was relatively non-toxic, a second experiment was carried out in which a further six mice were treated with 300 μCi $^{90}$Y-TFM. Tumour size in the untreated control mice was between 0.08 and 0.31 cm$^3$ (mean, 0.16 cm$^3$) and for the group treated with 300 μCi (100 μg) $^{90}$Y-TFM 0.17–0.26 cm$^3$ (mean, 0.21 cm$^3$). Tumour growth and toxicity were evaluated as described above. Tumours in the six mice that were left untreated grew rapidly and reached a mean maximum size of 0.83 cm$^3$ by day 9 (Figure 7a). In the $^{90}$Y-TFM-treated group, tumours again increased in size initially and reached a mean maximum size of 0.33 cm$^3$ by day 6. After this the tumours regressed and were not measurable by day 48 (Figure 7b). One mouse relapsed from treatment with a measurable tumour at day 72 after treatment. This mouse was killed when its tumour reached 2.0 cm$^3$ by day 104. The remaining mice stayed tumour free until the termination of the experiment at 7 months after treatment.

As in the experiment with 220 μCi $^{90}$Y-TFM, all animals survived treatment. There was no significant weight loss in any of the mice which received $^{90}$Y-TFM compared with the untreated group. The white blood cell count (Figure 8) showed a drop in the count in the treated group compared with control mice. The lowest blood count was obtained on day 6 at a mean value of 4580 per mm$^3$ which is 51% of the mean value measured in the control group. However, blood cell count in the control group was rather variable which is reflected in the standard deviations. From day 6 onwards cell counts recovered in the treated group. It is apparent by comparing Figures 5b and 8 that treatment with 300 μCi $^{90}$Y-TFM was less toxic to bone marrow than 250 μCi $^{90}$Y-IgG.

To enable a further comparison of the therapeutic effects of $^{90}$Y-A33 IgG and TFM dose calculations were performed based on the biodistribution data (Figure 1). These calculations employed the traditional MIRD methodology (Loevinger *et al.*, 1991) applied to mice, in which the absorbed fraction for the beta-ray emissions was assumed to be one, and the dosimetric contribution from Bremsstrahlung negligible. The results (Table I) demonstrate that the dose received by the blood in TFM-treated animals is approximately 5-fold lower per μCi injected than that received by IgG-treated animals. Also, the dose received by tumour for TFM-treated animals is some 3.4-fold lower per μCi injected than IgG-treated animals. This leads to an improvement in tumour–blood ratios for TFM of approximately 1.5-fold. In an attempt to perform therapy studies with $^{90}$Y-A33 IgG at equivalent doses to blood and tumour to that seen with $^{90}$Y-A33 TFM at 300 μCi, therapy experiments were thus performed with treatment at 59 μCi and 87 μCi of $^{90}$Y-A33 IgG. A group of six mice of tumour sizes 0.15–0.64 cm$^3$ (mean 0.36 cm$^3$) were treated with 87 μCi $^{90}$Y-A33 IgG, and a further group with tumour sizes 0.17–0.45 cm$^3$ (mean, 0.30 cm$^3$) were treated with 59 μCi $^{90}$Y-A33 IgG. In addition, a further non-specific control group, tumour sizes 0.17–0.43 cm$^3$ (mean, 0.29 cm$^3$) were treated with 87 μCi $^{90}$Y-MOPC21 IgG and an untreated control group, tumour sizes 0.13–0.65 cm$^3$ (mean, 0.34 cm$^3$) were used. Results from this experiment are shown in Figure 9. Control animals showed rapid tumour growth as expected (Figure 9a) and treatment with 87 μCi $^{90}$Y-MOPC21 IgG resulted in only a very modest anti-tumour effect (Figure 9b). Treatment with 59 μCi $^{90}$Y-A33 IgG, which should result in the same blood dose as 300 μCi $^{90}$Y-A33 TFM, resulted in tumour growth delay and regression for a mean of 26 days before regrowth took place in all animals (Figure 9c). The nadir white blood cell count in this case was reduced to 60% of the control value after 6 days (Figure 8). Similarly, when treated with 87 μCi $^{90}$Y-A33 IgG, a dose equivalent in tumour dose to 300 μCi $^{90}$Y-A33 TFM, a mean tumour growth delay and regression time of 32 days was observed before regrowth took place in all animals (Figure 9d). This is clearly a less potent anti-tumour effect than seen with 300 μCi $^{90}$Y-A33 TFM where complete regressions were observed in five out of six mice treated.

### Discussion

The inability of radiolabelled antibodies to reach their target tumour in adequate quantities has been a major factor

**Figure 6** Tumour growth in (**a**) untreated controls and (**b**) mice treated with 220 $\mu$Ci $^{90}$Y-labelled TFM. Each mouse bearing a 14-day-old tumour xenograft received one i.v. injection on day 0.

limiting their efficacy in cancer treatment. Once they reach the target organ the antibodies must be distributed, transported across the microvasculature wall and through the interstitial spaces (Jain, 1989). A number of these criteria are determined not only by the morphology and properties of the tumour itself but also by the properties of the chosen antibody and its antigen distribution and expression. In the present study, tumour distribution of three anti-colon cancer antibodies in SW1222 xenografts was established by autoradiography. All of these antibodies showed distinctive behaviour and among them A33 showed impressive penetration not only out of the blood vessels but through the tumour tissue. A number of repeated studies were conducted with A33 and in all cases A33 showed homogeneous penetration into the tumour tissue. Homogeneity of the A33 antigen throughout the colon tissue may be an important factor in the pattern of distribution obtained (Welt *et al*., 1994). The anti-CEA antibody A5B7 moved out of blood vessels effectively but did not travel far from the vessels into the tumour tissue. Similar observations have been reported for A5B7 in an LS174T xenograft system (Boxer *et al*., 1994). The difference in the distribution of the two antibodies within the tumour is quite distinct in spite of the fact that immunohistochemistry has shown a similar pattern of reactivity between A5B7 and A33 in colon cancer tissue (Boxer *et al*., 1994). B72.3 also demonstrated only limited penetration into the xenograft tumour tissue.

Although autoradiography experiments were non-quantitative, detailed examination of the tissues allowed preliminary conclusions to be drawn. A33 IgG penetrated tumour tissue extremely effectively and the derived A33 TFM appeared to penetrate even more rapidly with a more homogeneous distribution at 3 h. This may be significant for radioimmunotherapy with short half-life isotopes as much of the tumour dose is delivered at early times (Yorke *et al*., 1991). Considering previous work suggesting that tumour penetration is size dependent (Yokota *et al*., 1992) and that TFM and IgG have approximately the same molecular weight, the

**Figure 7** Tumour growth in (**a**) untreated controls and (**b**) mice treated with 300 $\mu$Ci $^{90}$Y-labelled TFM. Each mouse bearing a 14-day-old tumour xenograft received one i.v. injection on day 0.

more rapid penetration of TFM is unexpected and suggests that other properties of the molecule such as shape, charge or flexibility may also be important in achieving rapid penetration.

The overall biodistribution of $^{90}$Y-A33 demonstrated localisation to tumour with levels accumulating up to 35.9±5.25% ID g$^{-1}$ at 144 h after antibody injection. $^{90}$Y-A33 appeared to remain in the tumour as the blood concentration fell over the time of the experiment, a phenomenon not reported previously with other antibodies. It is possible that the pattern of tumour uptake reflects antibody internalisation into the tumour cells and the subsequent retention of the macrocycle-chelated isotope. There was little or no accumulation of $^{90}$Y-A33 in non-specific tissues emphasising the stability of the $^{90}$Y−12N4 macrocycle complex reported previously (Harrison *et al.*, 1991; DeNardo *et al.*, 1994). Colon levels of activity were low. However, it should be remembered that any cross-reaction with human colon would not be seen as the antibody is unlikely to cross-react with the equivalent mouse antigen. Immunohistochemistry studies have demonstrated some cross-reaction of A33 with normal human colon tissue, although clinical studies with radioiodinated A33 have not revealed any associated toxicity (Welt *et al.*, 1994). $^{90}$Y-TFM cleared much faster from the blood compared with IgG leading to lower tumour levels of 12.9±4.60% ID g$^{-1}$ obtained by 24 h after antibody injection. Again the tumour level remained high for a long period, only falling at 96 h despite almost complete removal of $^{90}$Y-TFM from the blood at this time. Among the normal tissues, kidney had the highest level of activity (6.40±0.45% ID g$^{-1}$ at 24 h), although this cleared much more rapidly than the tumour levels of activity. Accumulation did not occur in the kidney suggesting clearance through this organ. The route of clearance of TFM from the animal is not fully defined at present as we have not carried out full mass balance analysis

**Figure 8** Comparison of white blood cell count in control mice (○), mice treated with 300 μCi $^{90}$Y-TFM (▲), mice treated with 59 μCi $^{90}$Y-IgG (■) and mice treated with 87 μCi $^{90}$Y-IgG (□). Each point represents the mean and error bars represent the standard error ($n = 6$).

of the injected material. However, we have carried out whole body clearance studies with TFM labelled with $^{111}$In, as these studies cannot be carried out with $^{90}$Y owing to absorption of the beta radiation by the body. These studies reveal that clearance from the whole body parallels that from the blood (P Antoniw *et al.*, unpublished data), indicating that there is no deposition of activity in other tissues not examined in the biodistribution data presented.

The unexpectedly rapid blood clearance of TFM has recently been reported for another antibody, chimeric B72.3 labelled with $^{90}$Y using the same methodology (King *et al.*, 1994). These findings with A33 thus confirm that the normal tissue biodistribution pattern obtained is a consequence of the nature of the cross-linked antibody fragment, rather than a property of one individual antibody. The rapid blood clearance of TFM is of particular interest as it has the same molecular weight as IgG, and it may be that the Fc region which is lacking in TFM makes a contribution to the clearance pattern of IgG, Another possibility is that the nominal molecular weight cut-off for clearance of proteins through the kidney (Maack *et al.*, 1979) is not reliant solely on size but also on other properties of the protein such as shape, flexibility or charge in a similar way to the effect on tumour penetration. The lack of high level accumulation of $^{90}$Y in the kidney is unusual for antibody fragments labelled with metallic isotopes and overcomes a previous limitation to radioimmunotherapy with such fragments (seen for example by Sharkey *et al.* 1990; Junghans *et al.*, 1993). Studies with a similar cross-linked antibody tri-Fab have also been reported by Schott *et al.* (1993) with $^{105}$Rh-labelled tri-Fab derived from the anti-TAG 72 antibody CC49. They also concluded that *in vivo* their tri-Fab had blood clearance properties intermediate between IgG and $F(ab')_2$.

Although a very attractive nuclide for therapy, radioimmunotherapy using $^{90}$Y has previously been limited owing to the relatively poor chelators in use. Washburn *et al.* (1991) recorded colorectal carcinoma xenograft regression of approximately 40 days with 200 μCi $^{90}$Y-labelled CO17-1A. A repeated dose of 150 μCi proved fatal to the mice. Prolongation of the survival of nude mice bearing colon carinoma xenografts by administration of 120 μCi of $^{90}$Y-ZCE025 (anti-CEA antibody) has also been reported (Hyams *et al.*, 1989). The median survival was doubled in comparison with the mice treated with the radiolabelled non-specific antibody. These LS174T tumours were grown intraperitoneally (i.p.) and the labelled antibody was also administered i.p. only 7 days after the cell inoculation. In the studies reported here with A33 the subcutaneous tumours were well

**Table I** Tissue dose per μCi injected (cGy) calculated from biodistribution data presented Figure 1

| *Tissue* | *$^{90}$Y-IgG* | *$^{90}$Y-TFM* |
|---|---|---|
| Blood | 12.2 | 2.4 |
| Muscle | 0.7 | 0.4 |
| Bone | 1.4 | 1.1 |
| Lung | 3.7 | 1.4 |
| Liver | 4.9 | 4.4 |
| Spleen | 6.0 | 2.8 |
| Kidney | 4.5 | 7.6 |
| Colon | 1.0 | 0.8 |
| Tumour | 50.7 | 14.7 |
| Tumour–blood ratio | 4.2 | 6.1 |

established and growing before the $^{90}$Y-labelled antibody was administered i.v. and these tumours were eradicated with a single dose of either the IgG (250 μCi) or TFM (300 μCi). Only a slightly higher dose of TFM was required, which was at the same time less toxic to the bone marrow than the IgG.

Interesting information was obtained from the histology of the subcutaneous sites of tumour implantation in mice cured with $^{90}$Y-A33. The sites from the IgG-treated mice had small pockets of quiescent tumour cells visible among the necrotic and fibrotic areas. Examination of the sites from the two mice cured with 220 μCi of TFM however, demonstrated no tumour cells present. The rapid penetration of TFM in the tumour tissue which was demonstrated by autoradiography may play an important role in the cell killing activity considering that $^{90}$Y has a short half-life (64 h). Recent clinical data comparing $^{131}$I-A5B7 IgG and $F(ab')_2$ suggests that rapid penetration into the tumour can result in higher levels of activity localised to tumour for fragments compared with IgG (Lane *et al.*, 1994).

A number of reports have described delayed tumour growth and complete remission of solid tumour transplants by using various $^{131}$I-labelled monoclonal antibodies (Goldenberg *et al.*, 1981; Cheung *et al.*, 1986; Esteban *et al.*, 1987; Senekowitsch *et al.*, 1989; Blumenthal *et al.*, 1989). In most of these studies complete remission was only achieved when antibody treatment was administered within 24 h of tumour transplantation or by injecting a lethal amount of radiation. Attempts at experimental therapies have also been reported with $^{131}$I-labelled fragments (Larson *et al.*, 1983; Blumenthal *et al.*, 1989; Buchegger *et al.*, 1989; Pedley *et al.*, 1993). Successful eradication of colon carcinoma xenografts in nude mice has been demonstrated with the use of $^{131}$I-labelled $F(ab')_2$ although a dose of 2200 μCi per mouse was required either as a single dose or fractionated over three administrations (Buchegger *et al.*, 1989, 1990).

Attempts at radioimmunotherapy with $^{90}$Y-labelled fragments have not previously been reported owing to the high levels of kidney accumulation seen with conventional Fab and $F(ab')_2$ fragments (Sharkey *et al.*, 1990). Also, therapy with $^{90}$Y-macrocycle-labelled antibody has not been reported previously. Recently, therapy of 7-day-old xenografts has been demonstrated with the anti-TAG 72 antibody CC49 labelled with $^{177}$Lu via a similar macrocyclic ligand to that used for $^{90}$Y here (Schott *et al.*, 1994). Therapy studies with this relatively long lived isotope (half-life, 6.7 days) resulted in potent anti-tumour effects although severe toxicity was observed at the curative (500 μCi) dose with the death of 5/9 mice treated.

Here we have shown that both $^{90}$Y-A33 IgG and TFM can be effective as therapeutic agents when $^{90}$Y is chelated through a stable macrocyclic ligand. A slightly higher dose of TFM was required to achieve cures but resulted in less toxicity. When attempts to match the dose received by the blood and tumour were carried out it was clear that superior therapeutic effects were seen with $^{90}$Y-A33 TFM. The reason for this is unclear. One argument is that the whole body dose

**Figure 9** Tumour growth in (**a**) untreated controls, (**b**) mice treated with 87 $\mu$Ci ($\mu$g) $^{90}$Y-labelled MOPC21, (**c**) mice treated with 59 $\mu$Ci ($\mu$g) and (**d**) 87 $\mu$Ci ($\mu$g) $^{90}$Y-labelled A33. Each mouse bearing a 14-day-old tumour xenograft received one i.v. injection on day 0.

resulting from the higher total $\mu$Ci amount of $^{90}$Y used with TFM plays a role. However, cures were not observed with similar $\mu$Ci amounts (250 $\mu$Ci) of the non-specific antibody MOPC21. A further explanation may lie in the faster penetration into tumour tissue seen for TFM compared with IgG as seen by autoradiography. Early times are particularly important during therapy with short half-life isotopes such as $^{90}$Y owing to the maximum dose rate to the cells being achieved early, and thus rapid tumour penetration may be a significant advantage.

Consideration of the tissue doses calculated and presented in Table I demonstrates that the normal tissue receiving the highest dose is blood for IgG and kidney for TFM. In the absence of high levels of bone uptake, blood dose in this model is considered a reasonable estimate of the dose delivered to bone marrow. Thus, although TFM has a lower uptake of activity in the kidney than many antibody fragments (Sharkey *et al.*, 1990), it is likely that the dose-limiting organ will be switched from bone marrow to kidney. Experience from external beam radiation therapy shows that kidney doses in excess of 1500 cGy result in a 5% complication probability. If one conservatively ignores the dose-rate effect then one may wish to limit the kidney dose to <1500 cGy. Therefore, the maximum tolerated activity can be calculated as 1500/7.6 = 197 $\mu$Ci, which would yield a maximum tumour dose of 2901 cGy with a blood dose of 473 cGy. Therapy with IgG on the other hand, would be limited by bone marrow toxicity. However, as bone marrow transplantation is now becoming more common to reduce the toxicity associated with radioimmunotherapy (Press *et al.*, 1993), it would theoretically be possible to increase the dose of IgG which could be administered. Without considering bone marrow toxicity, one could administer 333 $\mu$Ci of IgG for the same level of kidney complications predicted with 197 $\mu$Ci TFM, resulting in a tumoricidal dose of 16 900 cGy. To improve therapy with TFM it would obviously be desirable to reduce kidney levels of activity further. Recently, strategies for reducing kidney accumulation of antibody fragments have been described, which suggest some further improvement may be possible (Behr *et al.*, 1995).

Early attempts at cancer radioimmunotherapy in the clinic have met with only limited success. Clinical effects have been limited largely to radiosensitive and easily accessible tumours such as lymphoma with only limited benefit observed in colorectal cancer (Mach *et al.*, 1991). Several factors have limited more effective therapy including the penetration into the tumour mass, dose-limiting toxicity to the bone marrow and the inability to retreat owing to the generation of an immune response to the administered antibody. In this report we have attempted to address tumour penetration and the optimal form of the antibody for tumour accumulation with least toxicity to allow development of an effective radioimmunotherapeutic. We have also demonstrated the effectiveness of this agent in the mouse xenograft model system. Great care should be taken in the interpretation of mouse xenograft therapy studies owing to the well-known differences in therapeutic efficacy observed between mice and humans (Buchsbaum, 1995; Knox, 1995). However, it seems likely that the benefits observed from stable chelation of $^{90}Y$ and effective tumour penetration may result in improved prospects for clinical therapy.

Another major problem with radioimmunotherapy in man is the generation of a patient immune response to the administered murine antibody which prevents repeat treatments. To address this problem we have constructed humanised (CDR-grafted) versions of A33 and A33 TFM (King *et al.*, 1995) which are currently being evaluated in clinical studies. Clinical studies with other antibodies have revealed that humanised antibodies do indeed reduce the immunogenicity of administered antibody (Stephens *et al.*, 1995) and suggest that repeat therapy will be a realistic possibility.

**Acknowledgements**
We would like to thank Phil Jupp of American Cyanamid, Gosport, UK for synthesis of the CT998 tri-maleimide cross-linker and A Millican, K Millar and B Boyce of Celltech for synthesis of the CT77 reagent. We would also like to thank Marion Dorning for assistance with animal experiments.

### References

ADAM T. (1989). Radioiodination for therapy. *Ann. Clin. Biochem.*, **26**, 244-245.

BADGER CC. (1990). Bone marrow toxicity for $^{131}I$-labelled antibodies. *Antibody Immumoconj. Radiopharm.*, **3**, 281-287.

BARENDSWAARD EC, WELT S, SCOTT A, GRAHAM M AND OLD LJ. (1993). Therapy of human colon cancer transplants in nu/nu mice with $^{125}I$- and $^{131}I$-monoclonal antibody A33 (abstract 2844). *Proc. Am. Assoc. Cancer Res.*, **34**, 477.

BEHR TM, SHARKEY RM, JUWEID M, ANINPOT R, GRIFFITHS GL AND GOLDENBERG DM. (1995). Reduction of kidney uptake of radiolabelled monoclonal antibody (MAb) fragments: preclinical and initial clinical results. *J. Nucl. Med.*, **36**, 19P-20P.

BLUMENTHAL RD, SHARKEY RM, KASHI R AND GOLDENBERG DM. (1989). Comparison of therapeutic efficacy and host toxicity of two different $^{131}I$-labelled antibodies and their fragments in the GW-39 colonic cancer xenograft model. *Int. J. Cancer*, **4**, 292-300.

BOXER GM, ABASSI AM, PEDLEY RB AND BEGENT RHJ. (1994). Localisation of monoclonal antibodies reacting with different epitopes on carcinoembryonic antigen (CEA) – implications for targeted therapy. *Br. J. Cancer*, **69**, 307-314.

BUCHEGGER F, PFISTER C, FOURNIER K, PREVEL F, SCHREYER M, CARREL S AND MACH JP. (1989). Ablation of human colon carcinoma in nude mice by $^{131}I$-labeled monoclonal anti-carcinoembryonic antigen antibody $F(ab')_2$ fragments. *J. Clin. Invest.*, **83**, 1449-1456.

BUCHEGGER F, PELEGRIN A, DELALOYE B, BISCHOFF-DELALOYE A AND MACH JP. (1990). Iodine-131 labelled Mab $F(ab')_2$ fragments are more effective and less toxic than intact anti-CEA antibodies in radioimmunotherapy of large human colon carcinoma grafted in nude mice. *J. Nucl. Med.*, **31**, 1035-1044.

BUCHSBAUM DJ. (1995). Experimental radioimmunotherapy and methods to increase therapeutic efficacy. In *Cancer Therapy with Radiolabelled Antibodies*, Goldenberg DM (ed.) pp. 115-140, CRC Press: Boca Raton, USA.

BURAS RR, WONG JYC, KUHN JA, BEATTY BG, WILLIAMS LE, WANEK PM AND BEATTY JD. (1993). Comparison of radioimmunotherapy and external beam radiotherapy in colon cancer xenografts. *Int. J. Radiat. Oncol. Biol. Phys.*, **25**, 473-479.

CHEUNG NKV, LANDMEIER B, NEELY J, NELSON AD, ABRAMOWSKY C, ELLERY S, ADAMS RB AND MIRALDI F. (1986). Complete tumor ablation with iodine 131-radiolabeled disialoganglioside GD2-specific monoclonal antibody against human neuroblastoma xenografted in nude mice. *J. Natl Cancer Inst.*, **77**, 739-745.

COLCHER D, HORAN-HAND P, NUTI M AND SCHLOM J. (1981). A spectrum of monoclonal antibodies reactive with human mammary tumour cells. *Proc. Natl Acad. Sci. USA*, **78**, 3199-3203.

COLCHER D, MILENIC D, ROSELLI M, RAUBITSHEK A, YARRANTON GT, KING D, ADAIR J, WHITTLE N, BODMER M AND SCHLOM J. (1989). Characterization and biodistribution of recombinant and recombinant/chimeric constructs of monoclonal antibody B72.3. *Cancer Res.*, **49**, 1738-1745.

COX JP, JANKOWSKI KJ, KATAKY R, PARKER D, BEELEY NRA, BOYCE BA, EATON MAW, MILLAR K, MILLICAN AT, HARRISON A AND WALKER C. (1989). Synthesis of a kinetically stable yttrium-90 labelled macrocycle-antibody conjugate. *J. Chem. Soc. Chem. Commun.*, **1989**, 797-798.

DENARDO GL, KROGER LA, DENARDO SJ, MIERS LA, SALAKO Q, KUKIS DL, FAND I, SHEN S, RENN O AND MEARES CF. (1994). Comparative toxicity studies of yttrium-90 MX-DTPA and 2-IT-BAD conjugated monoclonal antibody (BrE-3). *Cancer Suppl.*, **73**, 1012-1022.

ESTEBAN JM, SCHLOM J, MORTNEX F AND COLCHER D. (1987). Radioimmunotherapy of athymic mice bearing human colon carcinomas with monoclonal antibody B72.3: histological and autoradiographic study of effects on tumours and normal organs. *Eur. J. Cancer Clin. Oncol.*, **23**, 643-655.

GOLDENBERG DM, GAFFAR SA, BENNETT SJ AND BEACH JL. (1981). Experimental radioimmunotherapy of a xenografted human colonic tumor (GW-39) producing carcinoembryonic antigen. *Cancer Res.*, **41**, 4354-4360.

HARRISON A, WALKER CA, PARKER D, JANKOWSKI KJ, COX JPL, CRAIG AS, SANSOM JM, BEELEY NRA, BOYCE BA, CHAPLIN L, EATON MAW, FARNSWORTH APH, MILLAR K, MILLICAN TA, RANDALL AM, RHIND SK, SECHER DS AND TURNER A. (1991). The *in vivo* release of $^{90}$Y from cyclic and acyclic ligand-antibody conjugates. *Nucl. Med. Biol.*, **18,** 469–476.

HARWOOD PJ, BRITTON DW, SOUTHALL PJ, BOXER GM, RAWLINGS G AND ROGERS GT. (1986). Mapping epitope characteristics on carcinoembryonic antigen. *Br. J. Cancer*, **54,** 75–82.

HYAMS DM, ESTEBAN JM, BEATTY BG, WANEK PM AND BEATTY JD. (1989). Prolongation of survival of nude mice bearing human colon cancer. *Arch. Surg.*, **124,** 175–179.

JAIN RJ. (1989). Delivery of novel therapeutic agents in tumours: physiological barriers and strategies. *J. Natl Cancer Inst.*, **81,** 570–576.

JUNGHANS RP, DOBBS D, BRECHBIEL MW, MIRZADEH S, RAUBITSCHEK AA, GANSOW OA AND WALDMANN TA. (1993). Pharmacokinetics and bioactivity of 1, 4, 7, 10-tetra-azacyclododecane N,N',N'',N'''-tetraacetic acid (DOTA)-bismuth-conjugated anti-Tac antibody for α-emitter ($^{212}$Bi) therapy. *Cancer Res.*, **53,** 5683–5689.

KING DJ, TURNER A, FARNSWORTH APH, ADAIR JR, OWENS RJ, PEDLEY RB, BALDOCK D, PROUDFOOT KA, LAWSON ADG, BEELEY NRA, MILLAR K, MILLICAN TA, BOYCE B, ANTONIW P, MOUNTAIN A, BEGENT RHJ, SHOCHAT D AND YARRANTON GT. (1994). Improved tumour targeting with chemically cross-linked recombinant antibody fragments. *Cancer Res.*, **54,** 6176–6185.

KING DJ, ANTONIW P, OWENS RJ, ADAIR JR, HAINES AMR, FARNSWORTH APH, FINNEY H, LAWSON ADG, LYONS A, BAKER TS, BALDOCK D, MACKINTOSH J, GOFTON C, YARRANTON GT, MCWILLIAMS W, SHOCHAT D, LEICHNER P, WELT S, OLD LJ AND MOUNTAIN A. (1995). Preparation and preclinical evaluation of humanised A33 immunoconjugates for radioimmunotherapy. *Br. J. Cancer*, **72,** 1364–1372.

KNOX SJ. (1995). Overview of studies on experimental radioimmunotherapy. *Cancer Res.* (suppl.), **55,** 5832s–5836s.

LANE DM, EAGLE KF, BEGENT RHJ, HOPE-STONE LD, GREEN AJ, CASEY JL, KEEP PA, KELLY AMB, LEDERMAN JA, GLASER MG AND HILSON AJW. (1994). Radioimmunotherapy of metastatic colorectal tumours with iodine-131-labelled antibody to carcinoembryonic antigen: phase I/II study with comparative biodistribution of intact and $F(ab')_2$ antibodies. *Br. J. Cancer*, **70,** 521–525.

LARSON SM, CARRASQUILLO JA, KROHN KA, BROWN JP, MCGUFFIN RW, FERENS JM, GRAHAM MM, HILL LD, BEAUMIER PL, HELLSTROM KE AND HELLSTROM I. (1983). Localization of $^{121}$I-labeled p97-specific Fab fragments in human melanoma as a basis for radiotherapy. *J. Clin. Invest.*, **72,** 2101–2114.

LEE YCC, WASHBURN LC, SUN TTH, BYRD BL, CROOK JE, HOLLOWAY EC AND STEPLEWSKI Z. (1990). Radioimmunotherapy of human colorectal carcinoma xenografts using $^{90}$Y-labelled monoclonal antibody CO17-1A prepared by two bifunctional chelate techniques. *Cancer Res.*, **50,** 4546–4551.

LOEVINGER R, BUDINGER TF AND WATSON EE. (1991). MIRD *Primer for Absorbed Dose Calculations*. The Society of Nuclear Medicine Inc: New York.

LYONS A, KING DJ, OWENS RJ, YARRANTON GT, MILLICAN A, WHITTLE NR AND ADAIR JR. (1990). Site specific attachment to recombinant antibodies via introduced surface cysteine residues. *Protein Engin.*, **3,** 703–708.

MAACK T, JOHNSON V, KAU ST, FIGUEIREDO JA AND SIGULEM D. (1979). Renal filtration, transport, and metabolism of low-molecular-weight proteins: a review. *Kidney Int.*, **16,** 251–270.

MACH JP, PELEGRIN A AND BUCHEGGER F. (1991). Imaging and therapy with monoclonal antibodies in non-hematopoietic tumours. *Curr. Opin. Immunol.*, **3,** 685–693.

MOI MK, DENARDO SJ AND MEARES CF. (1990). Stable bifunctional chelates of metals used in radioimmunotherapy. *Cancer Res.* (suppl.), **50,** 789s–793s.

PEDLEY RB, BODEN JA, BODEN RW, DALE R AND BEGENT RHJ. (1993). Comparative radioimmunotherapy using intact or $F(ab')_2$ fragments of $^{131}$I anti-CEA antibody in a colonic xenograft model. *Br. J. Cancer*, **68,** 69–73.

PRESS OW, EARY JF, APPELBAUM FR, MARTIN PJ, BADGER CC, NELP WB, GLENN S, BUTCHKO G, FISHER D, PORTER B, MATTHEWS DC, FISHER LD AND BERNSTEIN ID. (1993). Radiolabelled antibody therapy of B-cell lymphoma with autologous bone marrow support. *N. Eng. J. Med.*, **329,** 1219–1224.

RICHMAN PI AND BODMER WF. (1988). Control of differentiation in human colorectal carcinoma cell lines: epithelial–mesenchymal interactions. *J. Pathol.*, **156,** 197–211.

SCHOTT ME, FRAZIER KA, POLLOCK DK AND VERBANAC KM. (1993). Preparation, characterization and *in vivo* biodistribution properties of synthetically cross-linked multivalent antitumour antibody fragments. *Bioconjugate Chem.*, **4,** 153–165.

SCHOTT ME, SCHLOM J, SILER K, MILENIC DE, EGGENSPERGER D, COLCHER D, CHENG R, KRUPER WJ, FORDYCE W AND GOECKLER W. (1994). Biodistribution and preclinical radioimmunotherapy studies using radiolanthanide-labelled immunoconjugates. *Cancer Suppl.*, **73,** 993–998.

SENEKOWITSCH R, REIDEL G, MOLLENSTADT, KRIEGEL H AND PABST HW. (1989). Curative radioimmunotherapy of human mammary carcinoma xenografts with iodine-131-labeled monoclonal antibodies. *J. Nucl. Med.*, **30,** 531–537.

SHARKEY RM, MOTTA-HENNESSY C, PAWLYK D, SIEGAL JA AND GOLDENBERG DM. (1990). Biodistribution and radiation dose estimates for yttrium and iodine labelled monoclonal antibody IgG and fragments in nude mice bearing human colonic tumor xenografts. *Cancer Res.*, **50,** 2330–2336.

SIEGAL JA, WESSELS BW, WATSON EE, STABIN MG, VREISENDORP HM, BRADLEY EW, BADGER CC, BRILL AB, KWOK CS, STICKNEY DR, ECKERMAN KF, FISHER DR, BUCHSBAUM DJ AND ORDER SE. (1990). Bone marrow dosimetry and toxicity for radioimmunotherapy. *Antibody Immunoconj. Radiopharm.*, **3,** 213–233.

STEPHENS S, EMTAGE S, VETTERLEIN O, CHAPLIN L, BEBBINGTON C, NESBITT A, SOPWITH M, ATHWAL D, NOVAK C AND BODMER M. (1995). Comprehensive pharmacokinetics of a humanized antibody and analysis of residual anti-idiotypic responses. *Immunology*, **85,** 668–674.

WASHBURN LC, YU-CHEN C, TAN HS, BYRD B, CROOK JE, STABIN MG AND STEPLEWSKI Z. (1988). Preclinical assessment of $^{90}$Y-labelled monoclonal antibody CO17-1A, a potential agent for radioimmunotherapy of colorectal carcinoma. *Nucl. Med. Biol.*, **15,** 707–711.

WASHBURN LC, LEE YCC, SUN TTH, BYRD BL, HOLLOWAY EC, CROOK JE AND STEPLEWSKI Z. (1991). Radioimmunotherapy of SW948 human colorectal carcinoma xenografts with repeated injections of $^{90}$Y-labelled monoclonal antibody CO17-1A. *Antibody Immunoconj. Radiopharm.*, **4,** 729–734.

WELT S, DIVGI CR, REAL FX, YEH SD, PILAR GC, FINSTAD CL, SAKAMOTO J, COHEN A, SIGURDSON ER, KEMENY N, CARSWELL EA, OETTGEN HF AND OLD LJ. (1990). Quantitative analysis of antibody localization in human metastatic colon cancer: studies with monoclonal antibody A33. *J. Clin. Oncol.*, **8,** 1894–1896.

WELT S, DIVGI CR, KEMENY N, FINN RD, SCOTT AM, GRAHAM M, GERMAIN JS, CARSWELL-RICHARDSE, LARSON SM, OETTGEN HF AND OLD LJ. (1994). Phase I/II study of iodine 131-labeled monoclonal antibody A33 in patients with advanced colon cancer. *J. Clin. Oncol.*, **12,** 1561–1571.

YOKOTA T, MILENIC DE, WHITLOW M AND SCHLOM J. (1992). Rapid tumor penetration of a single chain Fv and comparison with other immunoglobulin forms. *Cancer Res.*, **52,** 3402–3408.

YORKE ED, BEAUMIER PL, WESSELS BW, FRITZBERG AR AND MORGAN JR AC. (1991). Optimal antibody-radionuclide combinations for clinical radioimmunotherapy: a predictive model based on mouse pharmacokinetics. *Nucl. Med. Biol.*, **18,** 827–835.